# Supplementary material for: A window of opportunity for cooperativity in the T Cell Receptor
Source: Nat Commun. 2018 Jul 5;9:2618. doi: 10.1038/s41467-018-05050-6 (PMC6033938; doi:10.1038/s41467-018-05050-6)
Supplement: Supplementary file 1 — Supplementary Information [file 41467_2018_5050_MOESM1_ESM.pdf]

SUPPLEMENTARY NOTE 1

Mathematical model and parameter values

States of the model

In Supplementary Table 1 we summarize all the states of the model and the corresponding variables in the system of Ordinary Differential Equations summarized below. The equations capture the transitions corresponding to the rates depicted in Supplementary Fig. 2a and, also, the ligand-receptor dynamics. We have used the BioNetGen Markup Language (or, simply BNGL) to code the model and integrate it numerically[1] .

Variable	BNGL notation	Symbol in main text
x_1	R(state~R,p,p,p)	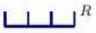
x_2	R(state~A,p,p,p)	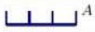
x_3	R(state~I,p,p,p)	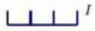
x_4	L(r,r,r)	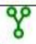
x_5	L(r!1,r,r).R(state~R,p!1,p,p)	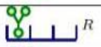
x_6	L(r!1,r,r).R(state~A,p!1,p,p)	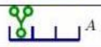
x_7	L(r!1,r,r).R(state~I,p!1,p,p)	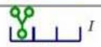
x_8	L(r!1,r,r).L(r!2,r,r).R(state~R,p!1,p!2,p)	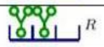
x_9	L(r!1,r,r).L(r!2,r,r).R(state~A,p!1,p!2,p)	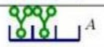
x_{10}	L(r!1,r,r).L(r!2,r,r).R(state~I,p!1,p!2,p)	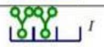
x_{11}	L(r!1,r!2,r).R(state~R,p!1,p!2,p)	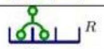
x_{12}	L(r!1,r!2,r).R(state~A,p!1,p!2,p)	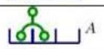
x_{13}	L(r!1,r!2,r).R(state~I,p!1,p!2,p)	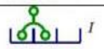
x_{14}	L(r!1,r,r).L(r!2,r,r).L(r!3,r,r).R(state~R,p!1,p!2,p!3)	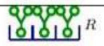
x_{15}	L(r!1,r!2,r).L(r!3,r,r).R(state~R,p!1,p!2,p!3)	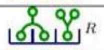
x_{16}	L(r!1,r,r).L(r!2,r,r).L(r!3,r,r).R(state~A,p!1,p!2,p!3)	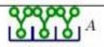
x_{17}	L(r!1,r!2,r).L(r!3,r,r).R(state~A,p!1,p!2,p!3)	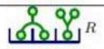
x_{18}	L(r!1,r,r).L(r!2,r,r).L(r!3,r,r).R(state~I,p!1,p!2,p!3)	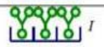
x_{19}	L(r!1,r!2,r).L(r!3,r,r).R(state~I,p!1,p!2,p!3)	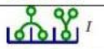
x_{20}	L(r!1,r!2,r!3).R(state~I,p!1,p!2,p!3)	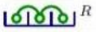
x_{21}	L(r!1,r!2,r!3).R(state~I,p!1,p!2,p!3)	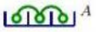
x_{22}	L(r!1,r!2,r!3).R(state~I,p!1,p!2,p!3)	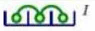
Wildcard example 1	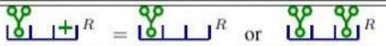	
Wildcard example 2	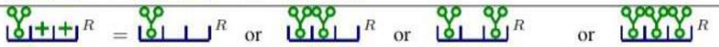	
Wildcard example 3	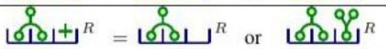	

Supplementary Table 1: Summary of BNGL symbols and their corresponding variable in the system of Equations below and cartoon representation used in the main text.

Mathematical equations

Despite the fact that the model has been written to respect detailed balance, we omit the direct transitions between the inhibited and the resting states, as these occur with respective on and off rates, $p_{on} \cdot q_{on}$ and $p_{off} \cdot q_{off}$. Due to the choice of parameters summarized in Supplementary Table 2, those two rates are negligible.

$$
\begin{aligned}
\frac{dx_1}{dt} &= k_{\rm off}x_5 - x_1(6k_{\rm on}x_4 + q_{\rm on}) + q_{\rm off}x_2 \\
\frac{dx_2}{dt} &= k_{\rm off}x_6 - x_2(6k_{\rm on}^{(A)}x_4 + p_{\rm on} + q_{\rm off}) + p_{\rm off}x_3 + q_{\rm on}x_1 \\
\frac{dx_3}{dt} &= k_{\rm off}x_7 - x_3(6k_{\rm on}x_4 + p_{\rm off}) + p_{\rm on}x_2 \\
\frac{dx_4}{dt} &= k_{\rm off}(x_5 + x_6 + x_7 + 2x_8 + 2x_9 + 2x_{10} + 3x_{14} + x_{15} + 3x_{16} + x_{17} + 3x_{18} + x_{19}) \\
&\quad -2x_4(k_{\rm on}(3x_1 + 3x_3 + 2x_5 + 2x_7 + x_8 + x_{10} + x_{11} + x_{13}) + k_{\rm on}^{(A)}(3x_2 + 2x_6 + x_9 + x_{12})) \\
\frac{dx_5}{dt} &= x_5(-(k_{\rm off} + 2k_{x,\rm on} + q_{\rm on})) + 2k_{\rm off}x_8 + 2k_{x,\rm off}x_{11} + 2k_{\rm on}x_4(3x_1 - 2x_5) + q_{\rm off}x_6 \\
\frac{dx_6}{dt} &= -x_6(k_{\rm off} + 2k_{x,\rm on} + p_{\rm on} + q_{\rm off}) + 2k_{\rm off}x_9 + 2k_{x,\rm off}x_{12} + 2k_{\rm on}^{(A)}x_4(3x_2 - 2x_6) + p_{\rm off}x_7 + q_{\rm on}x_5 \\
\frac{dx_7}{dt} &= -x_7(k_{\rm off} + 2k_{x,\rm on} + p_{\rm off}) + 2k_{\rm off}x_{10} + 2k_{x,\rm off}x_{13} + 2k_{\rm on}x_4(3x_3 - 2x_7) + p_{\rm on}x_6 \\
\frac{dx_8}{dt} &= -x_8(2(k_{\rm off} + k_{x,\rm on}) + q_{\rm on}) + 3k_{\rm off}x_{14} + 2k_{x,\rm off}x_{15} + 2k_{\rm on}x_4(2x_5 - x_8) + q_{\rm off}x_9 \\
\frac{dx_9}{dt} &= -x_9(2k_{\rm off} + 2k_{x,\rm on} + p_{\rm on} + q_{\rm off}) + 3k_{\rm off}x_{16} + 2k_{x,\rm off}x_{17} + 2k_{\rm on}^{(A)}x_4(2x_6 - x_9) + p_{\rm off}x_{10} + q_{\rm on}x_8 \\
\frac{dx_{10}}{dt} &= -x_{10}(2(k_{\rm off} + k_{x,\rm on}) + p_{\rm off}) + 3k_{\rm off}x_{18} + 2k_{x,\rm off}x_{19} + 2k_{\rm on}x_4(2x_7 - x_{10}) + p_{\rm on}x_9 \\
\frac{dx_{11}}{dt} &= k_{\rm off}x_{15} - x_{11}(2k_{x,\rm off} + 2k_{\rm on}x_4 + \tilde{q}_{on}) + 2k_{x,\rm on}x_5 + q_{\rm off}x_{12} \\
\frac{dx_{12}}{dt} &= k_{\rm off}x_{17} - x_{12}(2k_{x,\rm off} + 2k_{\rm on}^{(A)}x_4 + p_{\rm on} + q_{\rm off}) + 2k_{x,\rm on}x_6 + p_{\rm off}x_{13} + \tilde{q}_{on}x_{11} \\
\frac{dx_{13}}{dt} &= k_{\rm off}x_{19} - x_{13}(2k_{x,\rm off} + 2k_{\rm on}x_4 + p_{\rm off}) + 2k_{x,\rm on}x_7 + p_{\rm on}x_{12} \\
\frac{dx_{14}}{dt} &= -x_{14}(3k_{\rm off} + q_{\rm on}) + 2k_{\rm on}x_4x_8 + q_{\rm off}x_{16} \\
\frac{dx_{15}}{dt} &= -x_{15}(k_{\rm off} + 2k_{x,\rm off} + q_{\rm on} + \tilde{q}_{on}) + 2k_{\rm on}x_4x_{11} + 2k_{x,\rm on}x_8 + x_{17}(q_{\rm off} + q_{\rm off}) \\
\frac{dx_{16}}{dt} &= -x_{16}(3k_{\rm off} + p_{\rm on} + q_{\rm off}) + 2k_{\rm on}^{(A)}x_4x_9 + p_{\rm off}x_{18} + q_{\rm on}x_{14} \\
\frac{dx_{17}}{dt} &= -x_{17}(k_{\rm off} + 2k_{x,\rm off} + p_{\rm on} + q_{\rm off} + q_{\rm off}) + 2k_{\rm on}^{(A)}x_4x_{12} + 2k_{x,\rm on}x_9 + p_{\rm off}x_{19} + x_{15}(q_{\rm on} + \tilde{q}_{on}) \\
\frac{dx_{18}}{dt} &= -x_{18}(3k_{\rm off} + p_{\rm off}) + 2k_{\rm on}x_4x_{10} + p_{\rm on}x_{16} \\
\frac{dx_{19}}{dt} &= -x_{19}(k_{\rm off} + 2k_{x,\rm off} + p_{\rm off}) + 2k_{\rm on}x_4x_{13} + 2k_{x,\rm on}x_{10} + p_{\rm on}x_{17} \\
\frac{dx_{20}}{dt} &= -x_{20}k_{\rm off} + k_{x,\rm off}x_{11} + \tilde{q}_{\rm off}x_{21} - \tilde{q}_{\rm on}x_{20} \\
\frac{dx_{21}}{dt} &= -x_{21}k_{\rm off} + k_{x,\rm off}x_{12} + \tilde{p}_{\rm off}x_{23} - \tilde{p}_{\rm on}x_{21} - \tilde{q}_{\rm off}x_{21} + \tilde{q}_{\rm on}x_{20} \\
\frac{dx_{22}}{dt} &= -x_{22}k_{\rm off} + k_{x,\rm off}x_{13} - \tilde{p}_{\rm off}x_{22} + \tilde{p}_{\rm on}x_{21}
\end{aligned}
$$

(

Supplementary Table 2: Summary of the differential equations related to the model.

As the system of equations has many variables and parameters, we can make some practical simplifications aimed to estimate the model parameters. To start with, we will assume that the rates of cross-linking do not change dramatically for different states. This is obviously not true, since in the active state there are individual TCRs in a favourable orientation for binding, we have changed the relative cross-linking on rates up to an order of

magnitude and the results (for the low values of concentrations used) do not change significantly. This is probably due to the fact that cross-linking is not the main limiting time scale in the problem. Mathematically, this means that

$$k_{x,\mathrm{on}}^{(R)} = k_{x,\mathrm{on}}^{(A)} = k_{x,\mathrm{on}}^{(I)}.$$

The same argument holds for the off rates. Other approximations deserve more detailed descriptions, so we discuss them in the subsequent subsections.

Summary of parameters

In Supplementary Table 3 we summarize the parameters of the model and the references from which they have been obtained (when derived from the Literature).

**Parameter**	**Value**	**Reference**
Number of receptors	3×10^{4}	[2]
Volume	50 μl	(this work)
Number of cells	2×10^{5}	(this work)
Density (ρ)	0.1-1000 nM	(this work)
Off rate (25°C), $k_{off}^{(R)} = k_{off}^{(A)} = k_{off}^{(I)}$	$2.2\times10^{-2}s^{-1}$	[3]
On rate (25°C), $k_{on}^{(R)} = k_{on}^{(I)} = 5\times10^{-4}\ k_{on}^{(A)}$	$3720\ M^{-1}s^{-1}$	[3], See Text
Off rate (37°C), $k_{off}^{(R)} = k_{off}^{(A)} = k_{off}^{(I)}$	$2.2\times10^{-2}s^{-1}$	[3]
On rate (37°C), $k_{on}^{(R)} = k_{on}^{(I)} = 5\times10^{-4}\ k_{on}^{(A)}$	$2040\ M^{-1}s^{-1}$	[3], See Text
Tetramer off rate (37°C), $k_{off,t}^{(R)} = k_{off,t}^{(A)} = k_{off,t}^{(I)}$	$1.8\times10^{-3}s^{-1}$	[4]
Tetramer on rate (37°C), $k_{on,t}^{(R)} = k_{on,t}^{(I)} = 5\times10^{-4}\ k_{on}^{(A)}$	$486486\ M^{-1}s^{-1}$	[4], See Text
Crosslinking off-rate, $k_{x,off}$	k_{off}	[5]
Crosslinking on-rate, $k_{x,on}$	$(3\ k_{off}^{3}\ k_{on,t}/\ k_{on}\ k_{off,t})^{1/2}$	[5]
Resting to Active rate, q_{on}	$10^{-2}s^{-1}$	See Text

Active to Resting, q_{off}	$25q_{on}$	See Text
Enhanced Resting to Active, q^{*}_{on}	$q_{on}^{1/n}$	See Text
Active to Resting, q^{*}_{off}	q_{off}	See Text
Scaling factor, n	≥3.0	Fig. S1D (center)
Free energy factor, δ	≥2.0	Fig. S1D (right)
Active to Inhibited, p_{on}	$10^{-2}s^{-1}$	See Text
Inhibited to Active, p_{off}	$p_{on}/660s^{-1}$	See Text

Supplementary Table 3: Summary of the model parameters.

Estimation of the cross-linking conformational change enhancement

In order to clarify our parameter selection, let us start with the simple reaction

$L + R \to C$.

In this case, we find the evolution of binding (namely, the concentration of the complex [*C*]) follows the ODE:

$$\frac{d[C]}{dt} = k_{\text{on}}[R] \cdot [L] - k_{\text{off}}[C]$$

At short times, [*C*]~0 and [*L*] ~ρ (ρ being the initial ligand concentration in solution). Thus, we can assume that

$$\frac{d[C]}{dt} \simeq k_{\text{on}}[R]\rho \Rightarrow [C](t) \simeq k_{\text{on}}[R]\rho t \qquad (1)$$

As the binding is measured at fixed time *t* in Fig. 5b (right), the plot of [*C*](*t*) vs ρ has a slope proportional to $k_{on \cdot \rho}$. In our case, the receptors are split in three categories, namely, Resting, Active and Inhibited. So, for the small concentrations at which our experiments are performed,

we can assume that the binding on different clusters (*Resting*, *Active*, and *Inhibited*) are independent events. Namely,

$$[C](t) \simeq \frac{k_{\mathrm{on}}^{(R)}[R]_R + k_{\mathrm{on}}^{(A)}[R]_A + k_{\mathrm{on}}^{(I)}[R]_I}{[R]_R + [R]_A + [R]_I} \quad (2)$$

where $[R]_{R,A,I}$ are, respectively, the number of receptors in each state. We can estimate those rates from the initial free energy landscape in Supplementary Fig. 2a.

Following the works in receptor allostery in Ref. [6], a plausible landscape can provide free energy differences among states on the order of 2 kcal/mol. So, let F_R, F_A and F_I the free energies of the resting, active and inhibited states, we assume F_A-F_R = 2 kcal/mol and F_R-F_I = δx2 kcal/mol, where we allow to increase δ≥1. In the Supplementary Figure 2b (right panel) we show that the location of the maximum of the active state (corresponding to the experimental maximum of APA 1/1) is robust (meaning, it falls within the observed 5-10 minute range) for δ≥2. Taking into account that the *Inactive* state is an ensemble of states with more degrees of freedom than the other two, this larger well depth is reasonable.

Going back to Eq. (2), we can then assume that the initial equilibrium state ratios (prior to the addition of ligand) are given by:

$$\frac{[R]_R}{[R]_A} = e^{(F_A - F_R)/k_B T} \simeq 25, \text{ and } \frac{[R]_R}{[R]_A} = e^{(F_I - F_A)/k_B T} \simeq 660 \quad (3)$$

Where k_BT=0.543 kcal/mol at T=0°C . Assuming that the clone with the C80G mutation has almost 0 active states, and using the slopes in Fig. 5b (right panel) up to concentrations of OVAp=200 nM, we can conclude that,

$$\text{if } k_{\mathrm{on}}^{(R)} = k_{\mathrm{on}}^{(I)} \text{ then } k_{\mathrm{on}}^{(A)} \simeq 2 \times 10^3 k_{\mathrm{on}}^{(R)}$$

<u>Estimation of the inter-state rates</u>

Following the same estimations for the landscape as in Sec. 1.4, we can reduce the number of free parameters. Thus, the rates q_{on} and q_{off} depend on the relative free energies between the well free energies (F_R and F_A) and the free energy *peak*, F_{AR} between them. Thus, assuming a local equilibrium hypothesis (S.R. De Groot and P. Mazur Non-equilibrium thermodynamics. Courier Corporation, 2013), we have the following relationships:

$$\frac{q_{\mathrm{off}}}{q_{\mathrm{on}}} = \frac{e^{(F_{AR}-F_A)/k_BT}}{e^{(F_{AR}-F_R)/k_BT}} = 25$$

where we have used the same estimations as in Eq. (3). Likewise,

$$\frac{p_{\mathrm{on}}}{p_{\mathrm{off}}} = 660.$$

This leaves us with only two free parameters, for instance, q_{on} and q_{off}. In order to reproduce the characteristic time of the APA 1/1 maximum we set them to

$$q_{\mathrm{on}} = p_{\mathrm{on}} = 10^{-2}$$

Finally, as we discussed in the main text, we assume that the landscape changes induced by the conformational change that follows cross-linking. In our experiments, these changes seem to be almost independent of temperature and type of ligand,. To quantify this change, we introduce a scaling factor (*n*) that is, consequently, assumed to be independent of temperature.

Mathematically,

$$\tilde{q}_{on} = q_{on}^{n} \quad (4)$$

References

1 Blinov, M. L., Faeder, J. R., Goldstein, B. & Hlavacek, W. S. BioNetGen: software for rule-based modeling of signal transduction based on the interactions of molecular domains. *Bioinformatics.* **20**, 3289-3291. (2004).

2 Castro, M. *et al.* Receptor Pre-Clustering and T cell Responses: Insights into Molecular Mechanisms. *Front Immunol.* **5:132.**, 10.3389/fimmu.2014.00132. eCollection 02014. (2014).

3 Alam, S. M. *et al.* Qualitative and quantitative differences in T cell receptor binding of agonist and antagonist ligands. *Immunity* **10**, 227-237. (1999).

4 Daniels, M. A. *et al.* Thymic selection threshold defined by compartmentalization of Ras/MAPK signalling. *Nature.* **444**, 724-729. (2006).

5 Stone, J. D. *et al.* Interaction of streptavidin-based peptide-MHC oligomers (tetramers) with cell-surface TCRs. *J Immunol.* **187**, 6281-6290. doi: 6210.4049/jimmunol.1101734. (2011).

6 Gunasekaran, K., Ma, B. & Nussinov, R. Is allostery an intrinsic property of all dynamic proteins? *Proteins.* **57**, 433-443. (2004).

SUPPLEMENTARY FIGURES

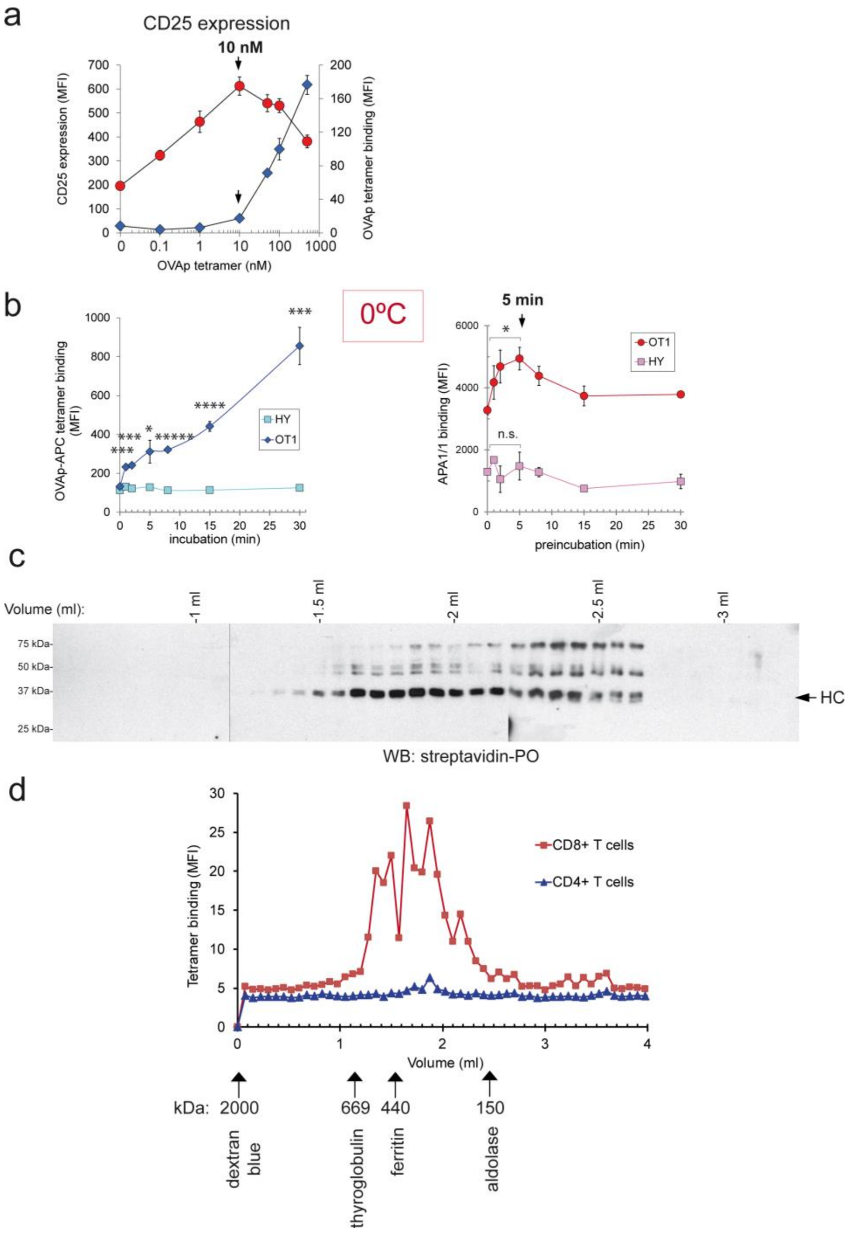

Supplementary Figure. 1. Specificity of the optimum binding time detected by APA1/1 staining. a, OT-1 CD8+ T cells were incubated at 37°C with the indicated concentrations of OVAp tetramer for 24 hours and stained with anti-CD25 and analyzed by flow cytometry**.** The MFI for CD25 staining is shown as red circles and the MFI of tetramer binding as blue diamonds. **b,** CD8+ T cells from OT-1 or HY female mice were incubated at different times at 0°C with 1 nM OVAp-APC tetramer, fixed, permeabilized and then stained with the APA1/1 mAb and analysed by flow cytometry. The MFI for OVAp-APC tetramer staining is shown on the left panel as blue diamonds for OT-1 cells and blue squares for HY cells. The MFI for APA1/1 staining is shown on the right panel as red circles for OT-1 cells and purple squares for HY cells. **c,** Fractions of OVAp-APC tetramer separated by gel filtration chromatography were analysed for MHC-I heavy chain (HC) content by SDS-PAGE and western blot with streptavidin-peroxidase. **d,** Binding to OT-1 CD8+ T cells of fractions generated be gel filtration in (c) and incubated for 40 min on ice. The positions of molecular weight markers used to calibrate the gel filtration column are indicated with arrows.

a

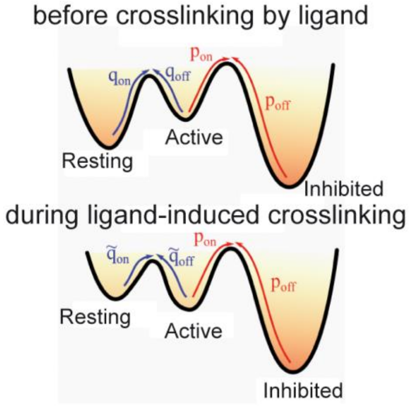

b

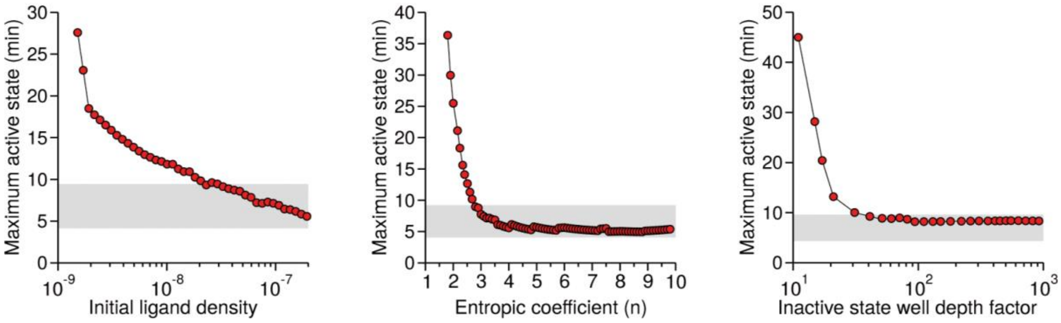

Supplementary Figure 2. Free energy landscape and robustness of the model. a, Schematic cartoon of the conjectured free energy landscape describing the three nanocluster states. After the addition of ligand and the subsequent induced crosslinking (bottom), the *Resting-Active* state transition becomes energetically favourable. **b,** Insensitivity of the location of the maximum within the experimental range (grey band) with respect to ligand concentration (left) and the main free parameters of the model: the degree of crosslinking-induced stabilization of the Active state (entropic factor), **n**, and the relative depth of the Inhibited state (free energy factor), δ. Although the model is not aimed at the detailed fitting of the experiments, the insensitivity of the model supports the three-state model for the conformational transitions within TCR nanoclusters.

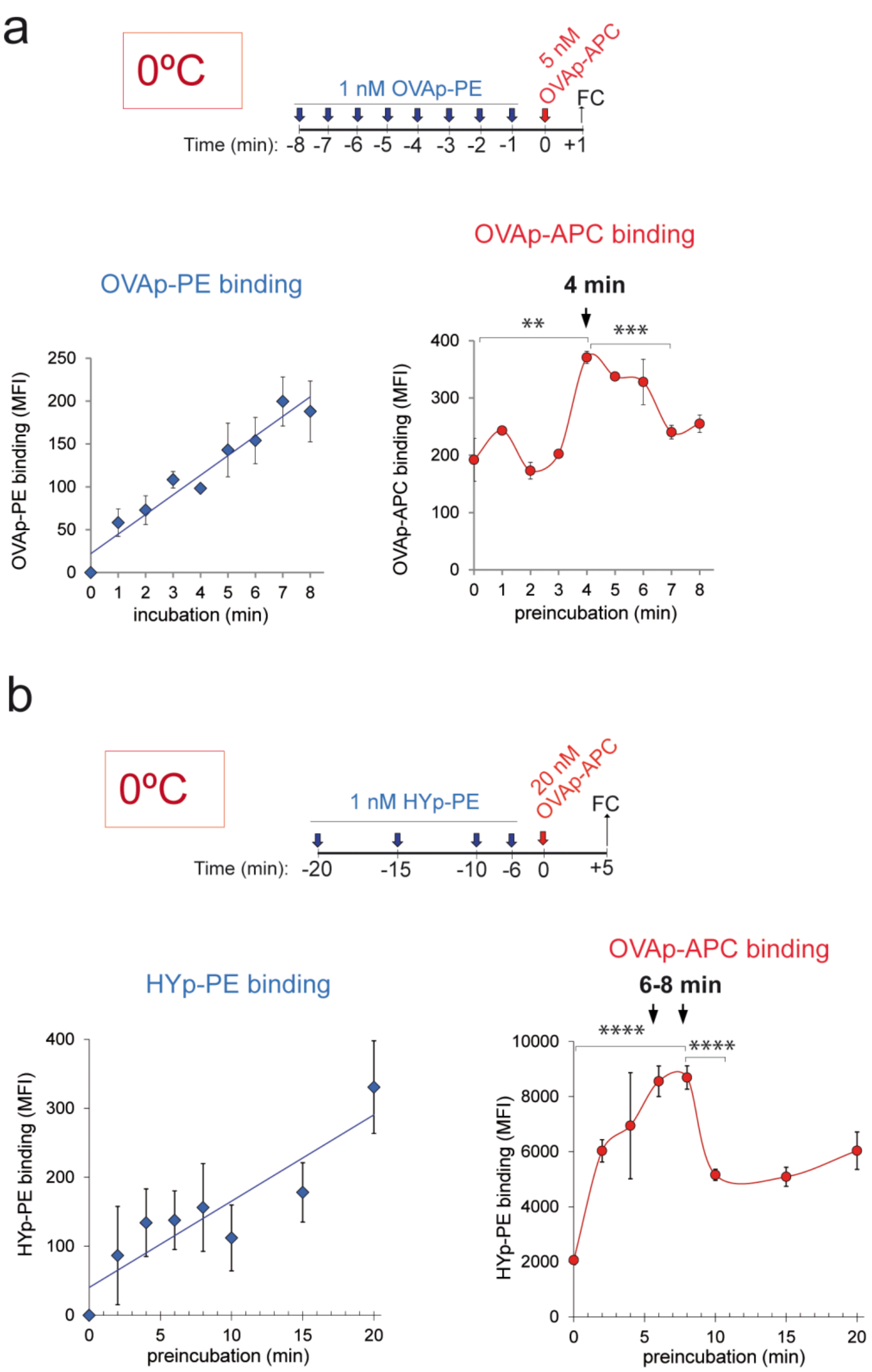

Supplementary Figure 3. TCR cooperativity manifested on ligand pMHC binding. a, CD8+ OT-1 T cells were preincubated on ice with 1 nM OVAp-APC tetramer for the indicated times and subsequently incubated with 5 nM of OVAp-PE tetramer for 1 additional minute. Cells were fixed and stained with anti-CD8 before analysis by flow cytometry and MFI for OVAp-PE and OVAp-APC calculation. **b,** Naïve OT-1xHY T cells were preincubated on ice with 5 nM HY-PE tetramer for the indicated times and subsequently incubated with 5 nM of OVAp-APC tetramer for 5 additional minutes. Cells were fixed and stained with anti-CD8 before analysis by flow cytometry and MFI calculation. All data represent the mean ± s.d. of triplicate datasets; ** *p*<0.005; *** *p*<0.0005; **** *p*<0.00005 (2-tail unpaired t-test).

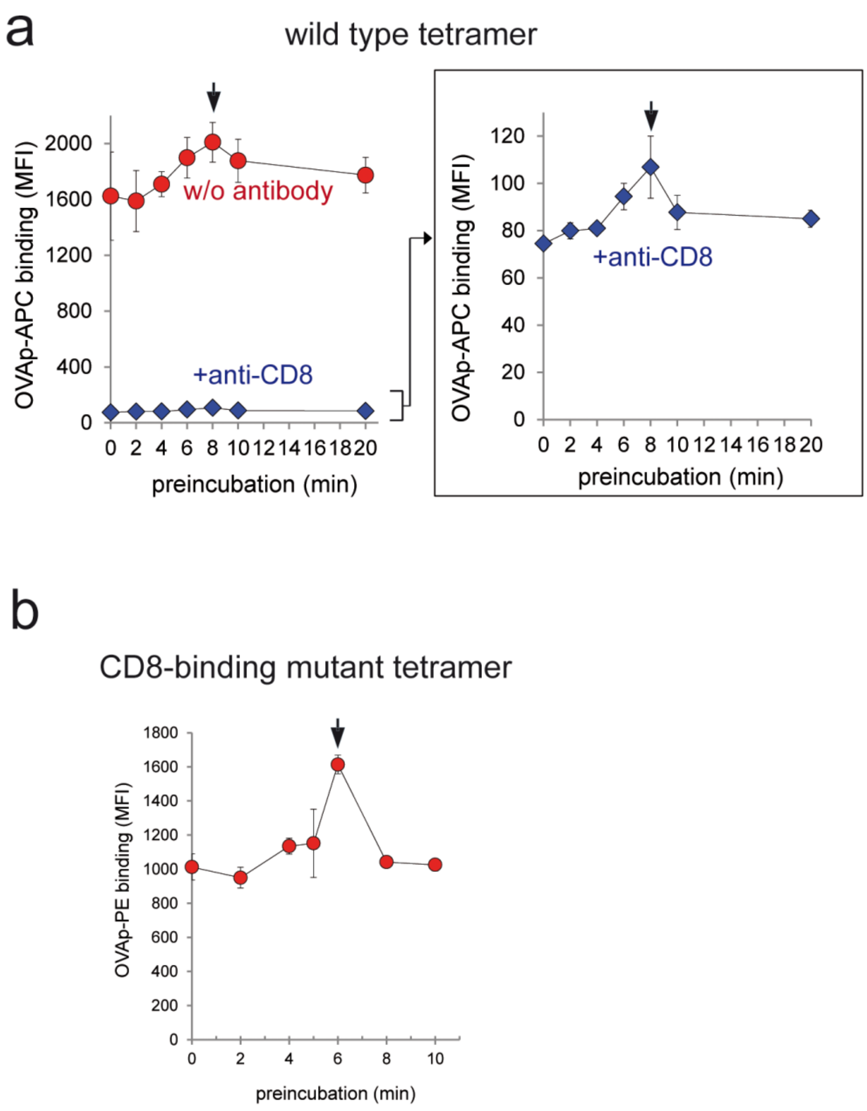

Supplementary Figure 4. Ligand pMHC binding cooperativity is independent of the coreceptor. a, CD8+ OT-1 cells pre-treated or not with a saturating anti-CD8 blocking antibody for 40 minutes were further preincubated with 1 nM OVAp-PE tetramer for the indicated times and subsequently incubated with 5 nM of OVAp-APC tetramer for 5 additional minutes; all incubations at 0°C. **b,** CD8+ OT-1 cells were preincubated at 0°C with 5 nM OVAp (CD8-blocked)-APC tetramer for the indicated times and subsequently incubated with 5 nM of OVAp-PE tetramer for 5 additional minutes. The OVAp (CD8-blocked) mutant cannot bind to CD8. All data represent the mean ± s.d. of triplicate datasets.

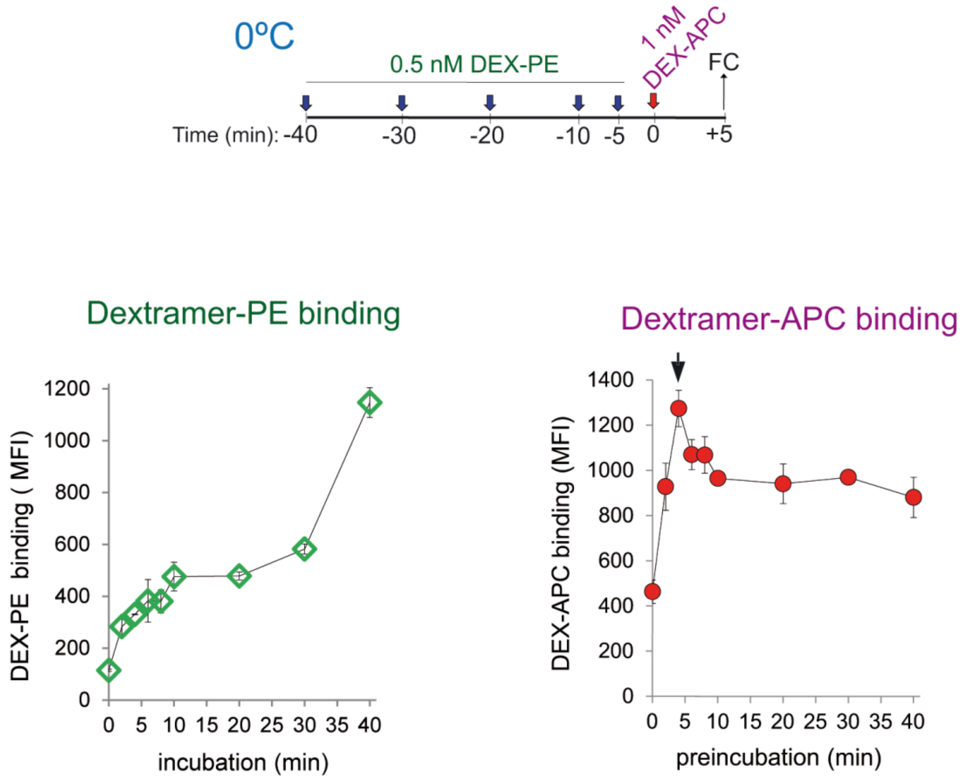

Supplementary Figure 5. Ligand binding cooperativity is also detected when using pMHC ligands of different geometry. CD8+ OT-1 T cells were preincubated at 0°C with 0.5 nM OVAp-PE dextramer for the indicated times and subsequently incubated with 1 nM of OVAp-APC dextramer for 5 additional minutes. Cells were fixed and stained with anti-CD8 before analysis by flow cytometry and MFI calculation. All data represent the mean ± s.d. of triplicate datasets.

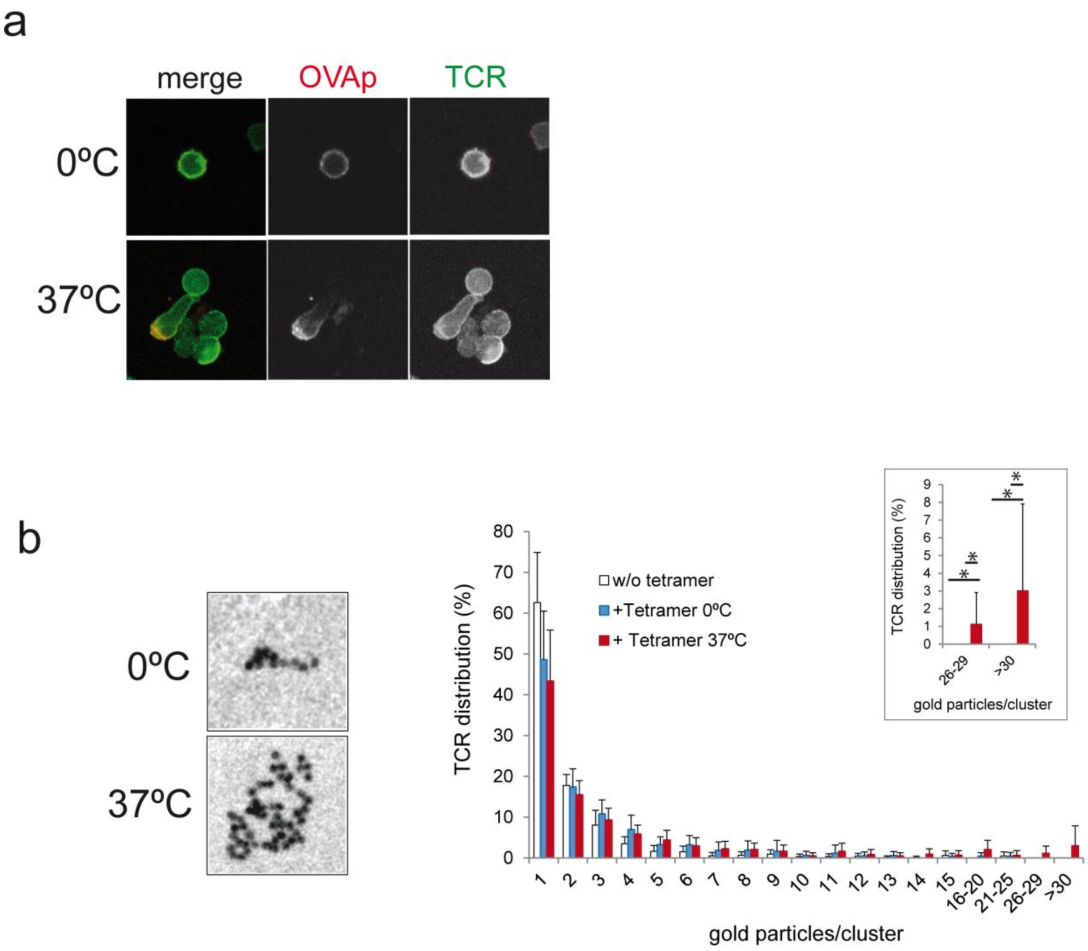

Supplementary Figure 6. Incubation with pMHC tetramer at 0°C does not induce TCR aggregation. a, CD8+ OT-1 T cells were attached to poly-*L*-lysine–coated coverslips and incubated with 5 nM OVAp-APC tetramer at either 0°C or 37°C. Before visualization by confocal microscopy, cells were fixed and stained with anti-TCRβ antibody H57. **b,** Illustrative electron microscopy images after label-fracture show TCR distribution on the membrane of OT-1 T cells incubated with 5 nM OVAp-APC tetramer for 5 min at either 0°C or 37°C (left). Quantification of TCR distribution according to the number of gold particles composing the clusters in non-incubated OT-1 T cells or incubated with 5 nM OVAp-APC tetramer at either 0°C or 37°C (right). Data shown represent the mean ± s.e.m. (n = 22 cells per condition). * $p<0.05$ (2-tail unpaired t-test). Values not marked with an asterisk were not significantly different ($p>0.05$).

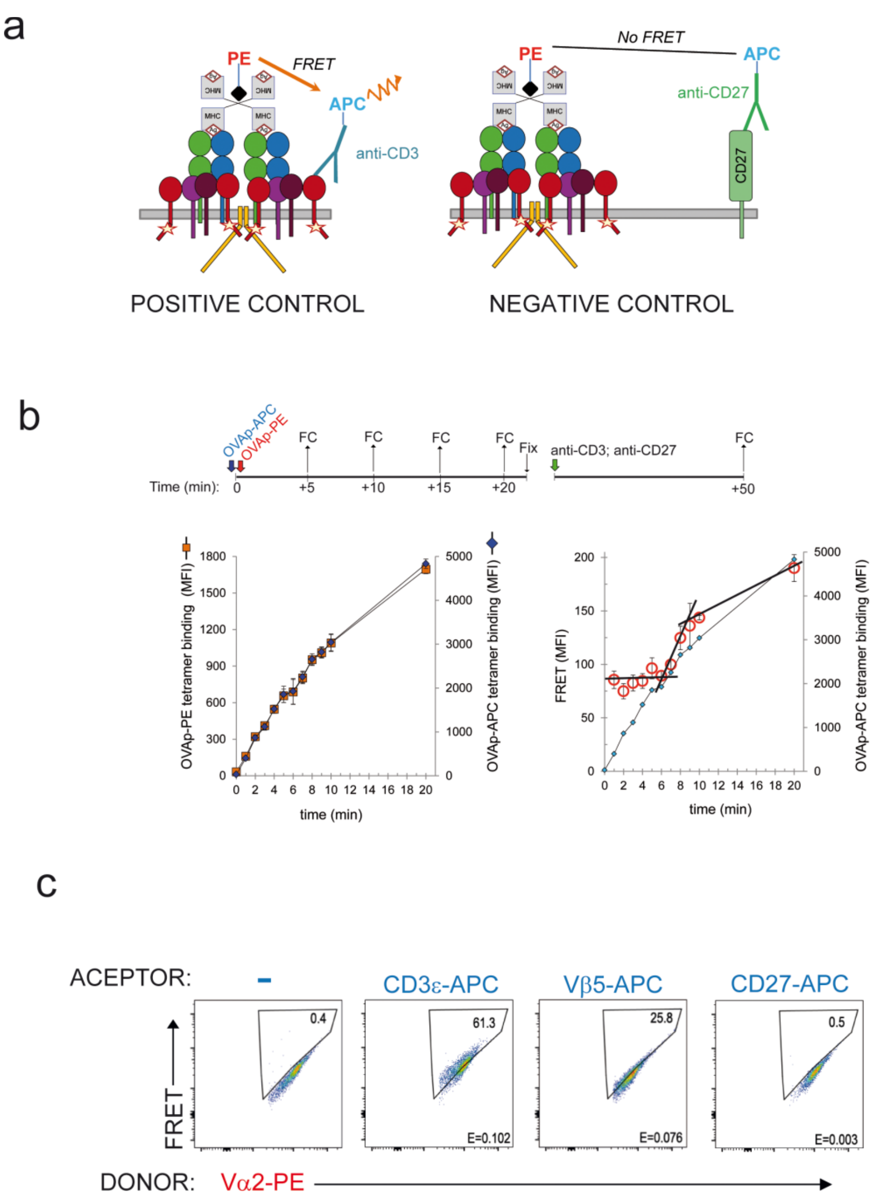

Supplementary Figure 7. Changes in the orientation or proximity of bound pMHC tetramers are detected in a time-dependent manner. a, Schematic of the experimental set-up. A positive FRET control was established by measuring FRET intensity between the OVAp-PE tetramer and an APC-labelled anti-CD3 antibody. The anti-CD3 was used at saturating concentrations. The tetramer binds TCRα/β and the anti-CD3 recognizes an epitope within the same TCR complex. A negative control was established using an APC-labelled anti-CD27 antibody as FRET acceptor. CD27 is not known to interact with the TCR. **b,** OT-1 T cells were simultaneously incubated on ice with 5 nM each of OVAp-PE and OVAp-APC tetramers for the indicated times and fixed. MFI for OVAp-PE and OVAp-APC are represented in the left panel, and MFI for FRET and OVAp-APC are represented in the right panel. Data shown represent the mean ± s.d. of triplicate datasets. **c,** FRET controls for different antibody combinations. OT-1 cells were labelled only with the donor (PE-conjugated antibody), with the donor and an anti-CD27-APC as acceptor or with both donor and acceptor. For each combination, the emission at 575 nm (PE-donor) and 675 nm (FRET) after excitation at 488 nm were recorded by flow cytometry. The polygonal region excludes the direct contribution of the donor in the optimal channel for FRET detection.

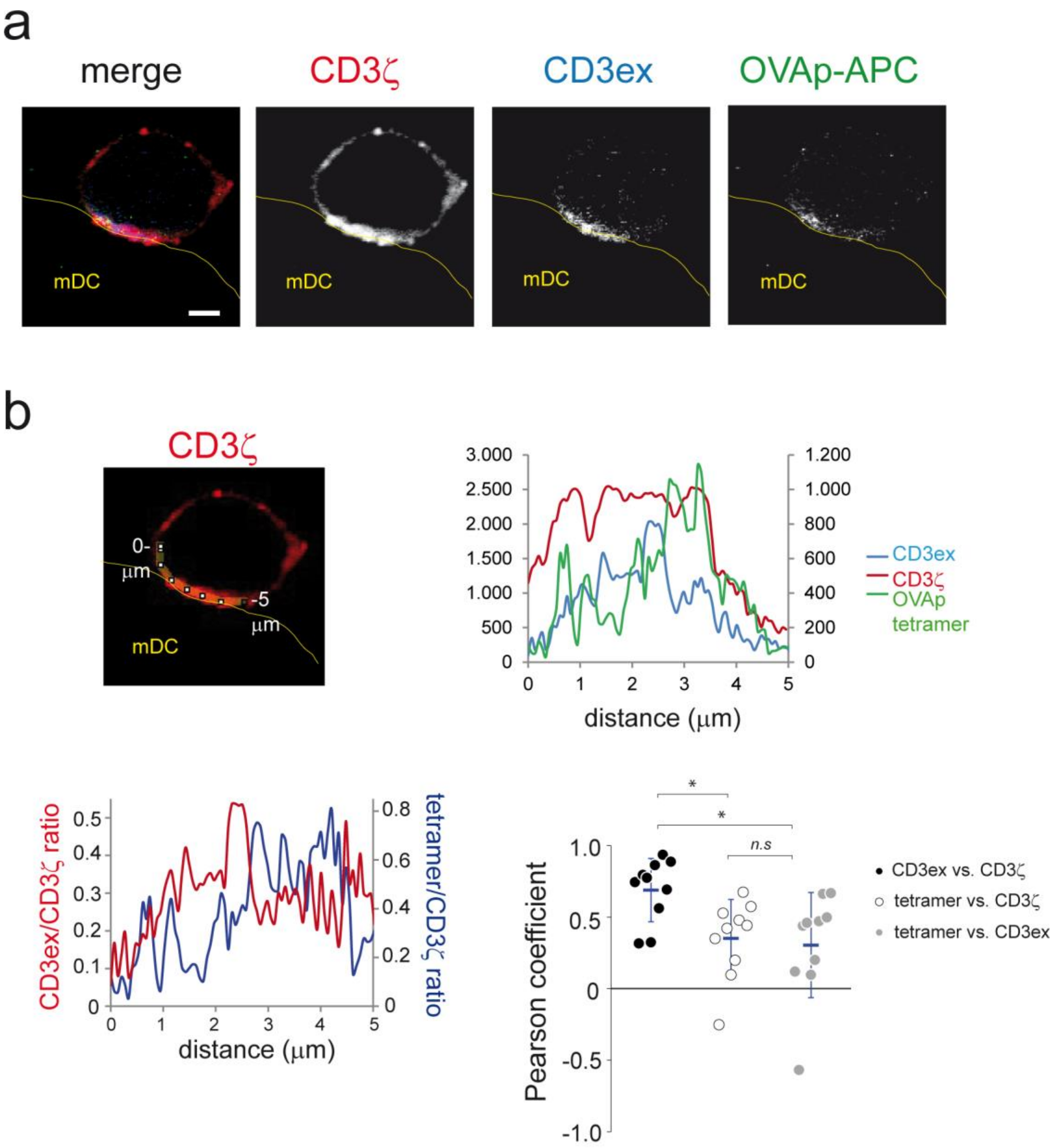

Supplementary Figure 8. A bulky anti-CD3-streptavidin complex is able to reach the centre of the IS. a, CD8+ T cells from OT-1 mice were purified by negative selection and incubated for 8 minutes at 37°C in the presence of mDCs attached to a coverslip and pre-loaded with OVA peptide. Subsequently, cells were incubated with 20 nM OVAp-APC and 5 μg/ml of a tetramerized Alexa555-labeled anti-CD3 for 5 additional minutes. The position of the antigen presenting cell (mDC) is marked in yellow type. Scale bar indicates 1 μm. **b,** Line scan drawn along the synaptic zone to show relative fluorescence intensity of CD3ζ, extracellular anti-CD3 (CD3ex) and OVAp-tetramer. Relative fluorescence intensity of CD3ζ, CD3ex and OVAp-tetramer is represented versus distance (μm). Ratio between the relative fluorescence intensities of CD3ex and CD3ζ, or OVAp-tetramer and CD3ζ, is represented versus distance (bottom left). Pearson correlation coefficients between CD3ex and CD3ζ, tetramer and CD3ζ, or CD3ex and CD3ζ intensity distributions for n= 10 cells is displayed (bottom right).
